# Validation of the Turkish version of European Organization for Research and Treatment of Cancer QLQ-OV28 ovarian cancer specific quality of life questionnaire

**DOI:** 10.4274/tjod.galenos.2020.26594

**Published:** 2020-04-06

**Authors:** Yeşim Akdemir, Çetin Cam, Nadiye Pınar Ay, Ateş Karateke

**Affiliations:** 1 Zonguldak Bülent Ecevit University Faculty of Medicine, Department of Obstetrics and Gynecology, Zonguldak, Turkey; 2 Uşak University Faculty of Medicine, Department of Obstetrics and Gynecology, Uşak, Turkey; 3 Marmara University Faculty of Medicine, Department of Public Health, İstanbul, Turkey; 4 İstanbul Medeniyet University Faculty of Medicine, Department of Obstetrics and Gynecology, İstanbul, Turkey

**Keywords:** Quality of life, ovarian cancer, EORTC

## Abstract

**Objective:**

To examine reliability and validity of the European Organization for Research and Treatment of Cancer (EORTC) QLQ-OV28 questionnaire into the Turkish language according to the instructions provided by EORTC.

**Materials and Methods:**

Ninety-seven patients who were diagnosed as having ovarian cancer and treated between January 2005 and June 2010 with an expected survival time of at least 3 months, were enrolled into the study. The exclusion criteria were diagnoses of any disease that could disrupt consciousness and concurrent malignancies. The EORTC QLQ-OV28 module was translated into Turkish by professional translators and physicians. The test–retest reliability of the Turkish version of the questionnaire was performed on 30 patients. Answers were scored according to the instructions provided by the EORTC. The total score was calculated as explained above and after scoring procedures, all subscale scores were linearly transformed to a 0-100 scale. All patients concomitantly completed the Spielberg State Trait Anxiety Inventory (STAI) and Beck Depression Inventory (BDI). Patients were analyzed in two groups: on-treatment and off-treatment groups consisted of patients who did and did not undergo chemotherapy or surgery within the last two months, respectively. The demographic data of all patients were recorded. EORTC QLQ-OV28 scores of both groups were compared. Correlations between EORTC QLQ-OV28 subscales and total score of BDI and STAI were analyzed.

**Results:**

For test-retest reliability, Spearman’s rho was 0.84 (p<0.001). The on-treatment group scored statistically significantly higher than the offtreatment group in peripheral neuropathies, attitude to disease and treatment, sexual function and other chemotherapy adverse effect subscales of the questionnaire. Correlations between EORTC QLQ-OV28 subscales and the total scores of BDI and STAI of the groups were statistically significant, except the sexual function subscale.

**Conclusion:**

The Turkish translated version of EORTC QLQ-OV28 module is a reliable, consistent, and a valid instrument for assessing the impact of treatment modalities on QoL among Turkish speaking women with ovarian cancer.

**PRECIS:** QoL of surviving patients has become an important goal of cancer research and Turkish translated version of EORTC QLQ-OV28 module can help physicians detect the physical and functional effects of disease, the treatment side effects and society/family reactions.

## Introduction

Ovarian cancer is a major gynecologic malignancy and the standard treatment is surgery followed by chemotherapy. Over the past 30 years, improvements in cure rates and length of survival have been observed. Five-year survival rates have been reported as high as 90% for patients diagnosed at stages of localized disease^(1)^. However, although the advances in the treatment of ovarian cancer have significantly improved survival, there remains a cost in terms of unpleasant adverse effects, which reduce the patient’s quality of life (QoL). This fact set another aspect in the cancer management beyond survival, and the QoL of surviving patients has become an important goal of cancer research. Inevitably, patient-reported QoL outcome instruments are now more important in cancer-related studies and in evaluating treatment results. An instrument that measures the QoL in these patients should cover the diagnosis of disease, the meaning of disease to the patient, the physical and functional effects of disease, the treatment adverse effects, and society/family reactions^(2)^. The European Organization for Research and Treatment of Cancer (EORTC) developed the EORTC QLQ-OV28, a symptom-specific questionnaire for use in ovarian cancer clinical trials to supplement the EORTC QLQ-C30, a cancer-specific core questionnaire^(3)^. The EORTC QLQ-OV28 module has been translated into many languages and validated in different populations^(4)^.

The current version of EORTC QLQ-OV28 module has been translated into 55 languages as of May 2019 around the world and has also been validated and tested in multicultural settings^(5)^. Although we obtained permission to translate the core questionnaire (OV28) from EORTC QOL Department in 2010, due to incomplete follow-up of permission process, an other Turkish translation of the module was accepted by EORTC QOL Department and is available for use. The current EORTC QLQ-OV28 Turkish module is only a translation without any clinical data and no published literature is available to examine various aspects of EORTC QLQ-OV28 module Turkish version translation and validation process^(5)^. Therefore, the aim of this study was to examine the reliability and validity of this questionnaire into Turkish language according to the instructions provided by EORTC by evaluating the clinical data of 97 patients^(6)^.

## Materials and Methods

### Questionnaire

The EORTC QLQ-OV28 module consists of 28 questions containing symptom scales [abdominal/gastrointestinal symptoms (GI) OV 31-37, peripheral neuropathy (PN) OV 41-43, other chemotherapy adverse effects (CH) OV 38-40, 44-47, hormonal/menopausal (HM) OV 48-49, attitude to disease and treatment (AT) OV 52-54, body image (BI) OV 50-51, and Sexual Function scale (SEF) OV 55-58]. Higher scores mean a poorer QoL, whereas for the items of SEF, OV 55-58, higher scores mean better QoL. Therefore, scores of SEF 55-58 need to be reversed during calculations of total scores. Two professional English-Turkish translators, who were not familiar with the EORTC QLQ-OV28, worked independently to produce the Turkish version of the questionnaire. At the first meeting, a common draft of the Turkish version was produced with a list of alternatives for the controversial items and response choices. At the second meeting, between the two translators and Turkish physicians with experience of health and QoL terminology, some revisions were made and a second draft was produced. Ten symptomatic women were asked to self-complete the second draft and then they were interviewed for possible ambiguous questions. At the third meeting, the final Turkish version was completed.

The study was approved by the ethics committee of the medical faculty of İstanbul Zeynep Kamil Women and Child Diseases Training and Research Hospital (approval number: 2019/139). The institutional ethics committee approved the study and informed consent was obtained from each participant. The Turkish version of the full questionnaire is available from the first author on request.

### Study population and data collection

Initially, a pilot study was conducted in order to evaluate the internal consistency and the test-retest reliability of the Turkish version of the questionnaire. Women completed the final version at their first visit in the oncology outpatient clinic of Zeynep Kamil Hospital (a tertiary referral teaching institution, Istanbul, Turkey), before meeting a physician. Questionnaires were printed in large fonts (16 font size) in order to be read and self-completed also by patients with poor eyesight. When a patient could not read or write, a relative or an accompanying helper of that patient assisted them in completing the questionnaire, if available. If not, support personnel, not familiar with the concepts of oncology and QoL, provided nondirective assistance to such patients. To measure the test-retest reliability of the final version of the questionnaire, a two-weeks’ test-retest analysis was used. Therefore, 30 women were asked to complete the questionnaire at their initial visit and repeat the procedure two weeks later in the same clinic. The responses of the two completed questionnaires were then analyzed using Spearman’s correlation test.

After the pilot study, patients who were diagnosed as having ovarian cancer and treated between January 2005 and June 2010 in the same institution, aged between 18-75 years, and with an expected survival time of at least 3 months, were enrolled into the study. The exclusion criteria were diagnoses of any disease that could disrupt consciousness and concurrent malignancies. The participants completed the questionnaire as described above. Answers were scored according to the instructions provided by the EORTC. The total score was calculated as explained above, and after scoring procedures, all subscale scores were linearly transformed to a 0-100 scale.

All patients concomitantly completed the Spielberg State Trait Anxiety Inventory (STAI) and the Beck Depression Inventory (BDI)^(7,8)^. STAI is a 40-item instrument that differentiates between the temporary condition of ‘state anxiety’ and the more general and long-standing quality of ‘trait anxiety.’ The STAI has been adapted into more than 40 languages^(7)^. To evaluate if EORTC QLQ-OV28 is a predicting instrument also for depression, the correlation with BDI was also assessed. The BDI is a 21-question multiple-choice self-report inventory that is one of the most widely used instruments for measuring the severity of depression. The evaluation of the psychometric properties and cut-off points of the BDI in a Turkish adult population was reported by Kapci^(8)^.

Patients were prospectively registered into the study and divided into two groups. The on-treatment group consisted of patients who were under chemotherapy or had surgery within the last two months. Patients of the off-treatment group had not received any treatment within the previous two months. The demographic data of all patients were recorded. The EORTC QLQ-OV28 scores of both groups were compared. Correlations between EORTC QLQ-OV28 subscales and total scores of the BDI and STAI were analyzed.

### Statistical analysis

The test-retest reliability was assessed by the Spearman’s correlation and Wilcoxon’s rank sum tests. A rho value of greater than 0.8 was considered as highly reliable^(9)^. Internal consistencies of the subscales were assessed using Cronbach’s alpha coefficient and item to other scale correlations. The content/face validity, which indicates whether the questionnaire makes sense to patients and experts, and whether all the important and relevant domains were included, was assessed by an expert panel that included two urogynecologists and one psychometrician. The levels of the missing data were used as an indicator of inappropriate questions^(10)^. Criterion validity, which describes how well the questionnaire correlates with existing standards^(10)^, was assessed by comparing the EORTC QLQ-OV28 scores with the scores of STAI and BDI. Spearman’s correlation coefficient was used for this purpose. All tests were performed using the Statistical Package for the Social Sciences (SPSS) for Windows 11.5. Values are given as percentage (%) or mean ± standard deviation (SD). Non-parametric tests were used for the analysis because of the non-normality of the data (Wilcoxon rank and Mann-Whitney U tests). The degree of statistical significance was set at <0.05 and all given p values were two-tailed.

## Results

A total of 97 patients who met the inclusion criteria were enrolled in the study. The mean age of the patients was 52.6±12.6 years. The on-treatment group comprised 39 patients and the off-treatment group consisted of 58 patients. The sociodemographic and clinical characteristics of the patients are detailed in Table 1.

The number of missing items was zero (0%). For the test-retest reliability, Spearman’s rho was 0.84 (p<0.001). The results of the internal consistency and item to other scale correlations were presented in Table 2.

Cronbach’s alpha values of all scales were higher than 0.70 and these results showed a high level of internal consistency. The on-treatment group scored higher (poorer QoL) than the off-treatment group, and the differences were statistically significant only in the PN, AT, CH, SEF subscales (Table 3). However, when the non-conditional items of SEF subscale (OV-55 and OV-56) were analyzed separately, the Cronbach’s alpha value was 0.93. The comparison of the scores of OV-55 and OV-56 of the on- and off-treatment groups were 96.15±10.44 and 82.47±19.60 (p<0.001). OV57 and OV58, conditional items to be answered only by sexually active women, were not statistically analyzed because only 50% (29/58) and 13% (5/34) of the patients answered these questions in the off- and on-treatment groups, respectively.

The total BDI and STAI scores were 10.4±8.8 and 77.2±19.9 in both groups, respectively. Correlations between EORTC QLQ-OV28 subscales and the total scores of BDI and STAI of the groups are presented in Table 4, where all of the correlations were statistically significant except the SEF subscale.

## Discussion

The results of this study show that the Turkish version of EORTC QLQ-OV28 has a high internal consistency and test-retest reliability. Overall, the on-treatment group showed poorer QoL than the off-treatment group. These results might be as a consequence of the heterogeneity of disease stage in the groups (high rates of advanced stages in the on-treatment group and high rates of borderline and early stages in the off-treatment group).

Similar results were reported in a study providing the validated Taiwan Chinese version of the EORTC QLQ-OV28^(4)^. Indeed, chemotherapy and/or surgery have an immediate negative effect on QoL and improvements in physical and functional well-being are likely to be observed at later intervals relative to earlier intervals after treatment^(11)^. Women previously treated for gynecologic cancer describe a range of psychosocial difficulties including depression and anxiety, and the most commonly described personal coping strategy is the use of positive thinking^(12)^. It is obvious that the severity and the discomfort of treatment-related symptoms may affect this attitude. In the present study, patients showed a significant correlation between their decreased QoL and depression and anxiety assessed by BDI and STAI questionnaires, respectively. The SEF subscale contains items pertaining to sexual function and two items (libido and extent of sexual activity) were asked of all patients and the remaining two questions were asked only to those who were sexually active. A significant number of women with gynecologic cancer are not sexually active. Furthermore, in the first phase of the development of the EORTC QLQ-OV28 module, items assessing sexual life had lower ratings than the other items^(3)^. In this study, especially in the on-treatment group, few women answered these items and, contrary to other subscales, significant differences and correlations were not observed in the SEF subscale. This finding was also observed in a study of validation of EORTC QLQ-OV28 into Taiwan Chinese and explained as a result of features of Chinese culture by the authors^(4)^. It should also be noted that in another randomized ovarian cancer study, the EORTC QLQ-OV28 module was designed without the use of these sexual function items^(13)^. Bajpai et al.^(14)^ also showed due to cultural taboos prevalent in India, people feel uncomfortable in talking about sexual behavior in general and a similar observation was noted when patients were asked to respond to question numbers 55-58 in the study of validation of EORTC QLQ-OV28 into Indian languages.

The inconsistency of these items with the module has led to the placement of this subscale at the end of the module, so that it could be omitted without interfering with the other presented items. The decision about neglecting or omitting should be made cautiously because the separate analysis of these non-conditional items of sexual function showed a high consistency and significant difference between the groups in the present study.

## Conclusion

Like the original questionnaire, the Turkish translated version of the EORTC QLQ-OV28 module is a reliable, consistent, and a valid instrument for assessing the impact of treatment modalities on QoL among Turkish-speaking women with ovarian cancer.

## Figures and Tables

**Table 1:**
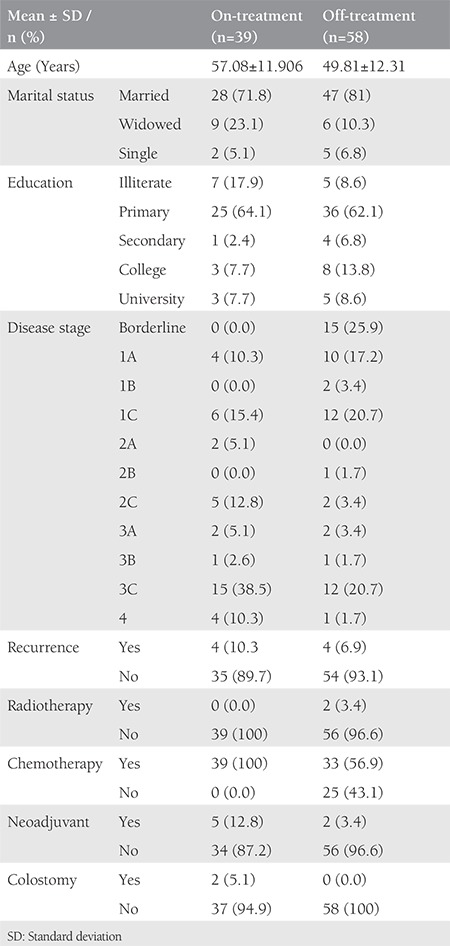
Sociodemographic and clinical characteristics of the patients

**Table 2:**
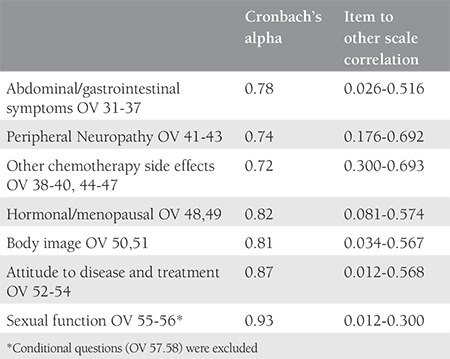
Internal consistency and item to other scale correlation

**Table 3:**
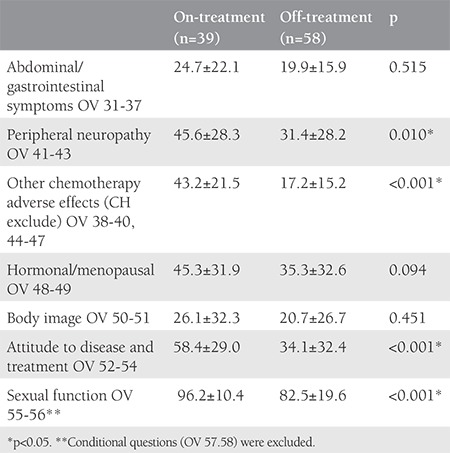
Comparison of quality of life scores of the European Organization for Research and Treatment of Cancer QLQ-OV28 between the groups (Mann-Whitney U test).

**Table 4:**
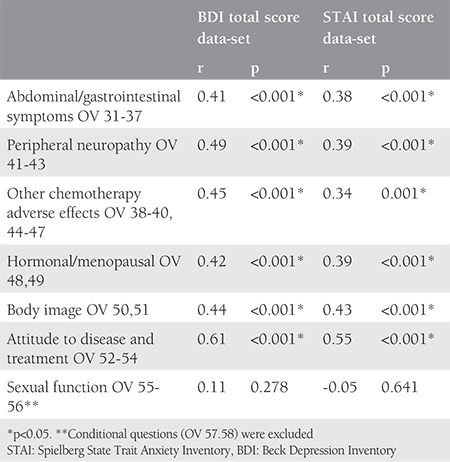
Correlations of European Organization for Research and Treatment of Cancer QLQ-OV28 subscales and total score of BDI and STAI between groups (Spearman’s correlation test).
